# LIFE: A metric for mapping the impact of land-cover change on global extinctions

**DOI:** 10.1098/rstb.2023.0327

**Published:** 2025-01-09

**Authors:** Alison Eyres, Thomas S. Ball, Michael Dales, Tom Swinfield, Andy Arnell, Daniele Baisero, América Paz Durán, Jonathan M. H. Green, Rhys E. Green, Anil Madhavapeddy, Andrew Balmford

**Affiliations:** ^1^ Department of Zoology, University of Cambridge, Cambridge, UK; ^2^ Conservation Research Institute, Department of Zoology, University of Cambridge, Cambridge, UK; ^3^ Department of Computer Science and Technology, University of Cambridge, Cambridge, UK; ^4^ UN Environment Programme World Conservation Monitoring Centre, Cambridge, UK; ^5^ Food and Agriculture Organization of the United Nations (FAO), Rome, Italy; ^6^ Key Biodiversity Areas Secretariat, c/o BirdLife International, Cambridge, UK; ^7^ Instituto de Ciencias Ambientales y Evolutivas, Facultad de Ciencias, Universidad Austral de Chile, Valdivia, Chile; ^8^ Department of Environment and Geography, Stockholm Environment Institute York, University of York, York, UK

**Keywords:** biodiversity metrics, extinction, persistence, land cover, restoration, habitat loss

## Abstract

Human-driven habitat loss is recognized as the greatest cause of the biodiversity crisis, yet to date we lack robust, spatially explicit metrics quantifying the impacts of anthropogenic changes in habitat extent on species’ extinctions. Existing metrics either fail to consider species identity or focus solely on recent habitat losses. The persistence score approach developed by Durán *et al*. (Durán *et al.* 2020 *Methods Ecol. Evol*. **11**, 910–921 (doi:10.1111/2041-210X.13427) represented an important development by combining species’ ecologies and land-cover data while considering the cumulative and non-linear impact of past habitat loss on species’ probability of extinction. However, it is computationally demanding, limiting its global use and application. Here we couple the persistence score approach with high-performance computing to generate global maps of what we term the LIFE (Land-cover change Impacts on Future Extinctions) metric for 30 875 species of terrestrial vertebrates at 1 arc-min resolution (3.4 km^2^ at the equator). These maps provide quantitative estimates, for the first time, of the marginal changes in the expected number of extinctions (both increases and decreases) caused by converting remaining natural vegetation to agriculture, and restoring farmland to natural habitat. We demonstrate statistically that this approach integrates information on species richness, endemism and past habitat loss. Our resulting maps can be used at scales from 0.5–1000 km^2^ and offer unprecedented opportunities to estimate the impact on extinctions of diverse actions that affect change in land cover, from individual dietary choices through to global protected area development.

This article is part of the discussion meeting issue ‘Bending the curve towards nature recovery: building on Georgina Mace's legacy for a biodiverse future’.

## 1. Introduction

Biodiversity loss is one of the greatest environmental challenges of our age, with declines associated with significant negative impacts on human wellbeing [1]. Tracking and mitigating these losses requires robust, spatially explicit biodiversity metrics for monitoring overall trends, identifying where conservation actions might be most effective, and measuring progress towards local-to-global biodiversity targets [2,3]. According to the IUCN Red List, agriculture and logging, the two main activities that drive land-use change, threaten 70 and 46% of terrestrial vertebrate species, respectively [4]. Moreover, land-use change looks set to remain the largest single threat for at least the next few decades [5–8] and is likely to interact with other threats to biodiversity such as climate change, underscoring the importance of biodiversity metrics that are linked directly with trends in land use [9].

While biodiversity is complex and multifaceted, metrics used in conservation arguably represent two primary motivations, as reflected in the targets of the Global Biodiversity Framework [10]: preventing species loss [11] and maintaining the integrity of ecosystems and their contributions to people [1]. In broad terms, such metrics typically comprise measures of spatial and temporal variation in extinction risk of species and intactness of ecosystems, respectively. Measures of extinction risk commonly incorporate features such as the number of species present in an area, their range sizes (and hence how important the area is for their persistence globally) and descriptions of how population sizes or ranges have changed or might do so. Intactness, on the other hand, describes anthropogenic impacts on biological communities, with declines in intactness taken to indicate biodiversity loss and reductions in ecosystem functions and associated ecosystem services.

To track variation in extinction risk or ecosystem intactness in relation to targets and to identify the likely positive or negative impacts of anthropogenic actions in different places, we suggest that metrics should:

1. Strive to be representative—geographically, taxonomically and in terms of habitat types. Given marked differences in the availability of data for different regions, taxonomic groups and habitat types, unrepresentativeness is a significant limitation of several metrics. For example, the Living Planet Index [12] uses data only for vertebrates, much of it from Europe and North America, though in this case substantial efforts are made to adjust statistically for differences in the coverage of different classes and regions [13]. The IUCN’s Species Threat Abatement and Restoration metric (STAR) [5] currently covers amphibians, birds and mammals. However, as STAR considers species of Least Concern to have zero extinction risk, it is currently unable to quantify the impact of land-use changes on those species and instead focuses on threatened and near-threatened species. Some other metrics, such as the Biodiversity Intactness Index (BII) [14], are based on data that are more representative taxonomically and by threat—in this case, measures of relative abundance of nearly 60 000 non-threatened as well as threatened plant and animal species.
2. Be comparable across space and direction of biodiversity change (that is, across gains and losses). Spatial comparability means that a given score for the metric in one location is equivalent in terms of the broad outcome of interest (extinction risk or ecosystem intactness) to the same value in any other location and that an area with twice that value is twice as important. Spatial comparability is essential when comparing actions in different locations—and so is of particular relevance in setting spatial priorities, in assessing the impacts of actors (e.g. international NGOs or corporations) who operate in different countries or in understanding the contribution of national activities towards global targets [2]. Metrics that treat all pristine habitats as of equal value—such as Mean Species Abundance (MSA) and the BII [14,15]—make it difficult to compare the impact of actions on habitats that differ markedly in the communities or ecosystem services they support. Directional comparability—where a score of *x* is equal and opposite to a score of −*x*—is essential when there is interest in identifying opportunities to mitigate damaging operations through remedial actions elsewhere—although, of course, where those actions involve habitat restoration, additional safeguards are necessary because of time lags and uncertainties in habitat recovery. Although the IUCN’s STAR metric identifies gains in biodiversity that could result from habitat restoration and threat abatement, it does not currently consider the impacts of continued habitat loss [5].
3. Be amenable to aggregation and disaggregation according to species, ecosystems and other factors. This can be useful where stakeholders are interested only in certain taxonomic groups, charismatic species or biomes and can allow for analyses of the impacts of particular threatening processes, as well as the sensitivity of observed patterns to unrepresentativeness of the underlying data. Furthermore, biodiversity metrics play a key role in halting biodiversity loss through raising awareness with the public and policymakers. Given this, it is important that such metrics are easy to understand and interpret [16]. Globally, aggregated metrics are often difficult to understand or relate to, but disaggregation can allow stakeholders to understand policy targets relating to both national and international commitments. Many metrics, however—such as MSA and the Sustainable Ecology and Economic Development frameworl(SEED) [15,17]—are not readily disaggregated, as species identity is not retained through computation.
4. Finally, to be useful in guiding real-world actions that vary in area, it is important that biodiversity metrics provide information that is scalable without the need for extensive additional analysis. If an action is larger than the grid size at which the metric is presented, can its impact be reliably estimated by using the scores for component grid cells—and likewise, does the score for a grid cell reliably indicate the value of action smaller than one grid cell? To what degree can published maps of metric scores be used, without rerunning the underlying algorithms, to assess the biodiversity impact of restoration or conversion actions that are much larger or smaller than the grid cells used to derive the maps? The importance of this is highlighted by the inclusion in the SMART targets (specific, measurable, achievable, relevant and time-bound) paradigm of ‘Measurable’, defined as ‘being able to assess progress towards the target using data already available or feasible to mobilize’ [18].

## 2. Conceptual basis of the Land-cover change Impacts on Future Extinctions (LIFE) metric

In this paper, we present a new global metric, which we term LIFE, which attempts to map for the first time the numbers of extinctions resulting from marginal losses and gains in the extent of natural habitats. LIFE is a global-scale progression of Durán *et al*.’s [19] persistence score approach and builds on a series of earlier foundational papers [20–24]. It is based on the following five fundamental assumptions and assertions around mapping anthropogenic extinction risks:

1. That it is useful to focus on quantifying likely human-caused extinctions. Extinctions of course arise naturally, but given that current extinction rates are roughly three orders of magnitude above background [25], here we look at changes in risks of extinction relative to extinctions in the absence of people. As all species eventually go extinct (or evolve into new species), it is important to note that LIFE is specifically concerned with human-driven extinctions arising from present-day actions that manifest over 100 years—a timescale that aligns with faunal relaxation times following anthropogenic habitat loss and with the IUCN Red List criteria [26,27].
2. That a species’ change in extinction risk as a result of human action depends on its current or future population size relative to that in the absence of people (hereafter its ‘original population’), as well as on its absolute population size. Absolutely small populations are of course at greater risk of extinction due to chance events [28], but we suggest that species that have had small population sizes through their evolutionary history are likely to have been selected to be more resilient to extinction at that size than other species [29]. Hence, reducing a species to half its original population size will have roughly the same effect on its extinction risk regardless of whether it was naturally abundant or scarce. LIFE does not concern itself with species that are nowadays more abundant than in their evolutionary past.
3. That a species’ risk of extinction within any specified timescale scales non-linearly with its current risk relative to the original population, with a given marginal decline having a small effect when a population is close to its original size but a much greater effect when a population has already been greatly reduced. The exact shape of this curve is not known and will presumably vary with a species’ life history, demography and ecology. Importantly, non-linearity means that metrics that instead assume linearity will tend to underestimate the impacts of population declines in already severely impacted species, and also that estimating contemporary impacts requires present-day population sizes to be expressed relative to original population sizes.
4. That in the first instance it is reasonable to focus on anthropogenic changes in habitat extent and quality, because these constitute the greatest current and future source of threat to terrestrial biodiversity [7,30,31] . Other threats, such as overexploitation [32] and invasive alien species [33], are also extremely important and will determine how far a species is able to occupy an area of suitable habitat, but they are poorly mapped at global scale [34], so their incorporation into worldwide area-based metrics is problematic (but see [5]).
5. That although species’ occupation of suitable habitats will vary with their ecology, with habitat condition, fragmentation, connectivity and so on, until these effects can be estimated separately for very many species, as a first step, it is useful to estimate land cover-mediated changes in relative extinction risk using changes in species’ Area of Habitat (AOH), again estimated relative to that in the absence of people. While AOH—defined conceptually as the habitat available to a species and in practice mapped as the intersection between a species’ range and its environmental preferences [35]—is of course an imperfect surrogate, it is for now the only measure of species’ distributions that is available for tens of thousands of species.

## 3. Developing the LIFE metric

LIFE takes as its starting point Durán *et al*.’s [19] persistence score. This uses species-specific distribution and habitat suitability information to estimate the consequences of marginal changes in land cover for the modelled probability that species will persist (i.e. avoid extinction), relative to their probability of persisting in the absence of anthropogenic habitat change (see §2). Duran *et al.* did not specify a time period. As LIFE is concerned with human-driven extinctions, we therefore assess the probability of extinction over 100 years—a timescale at which human-driven habitat loss occurs and biodiversity impacts have stabilized. Changes can be gains or losses of suitable habitat, with negative scores equal and opposite to positive ones. Uniquely, the persistence score also accounts explicitly for the likely non-linear relationship between habitat loss and changes in species’ probability of persistence, and considers the cumulative impact of habitat loss over the long term, rather than just recent changes. Both of these issues are overlooked in other metrics of extinction risk. Other approaches instead typically assume extinction risk only depends on contemporary change in AOH: that a 100 km^2^ loss of habitat for a species’ currently occupying 1000 km^2^ has the same effect regardless of whether in the absence of people it would occupy 1000 or 1 million km^2^ [36–38]. However, there is substantial evidence that the impacts of habitat loss on species extinction risk are typically cumulative and non-linear, with the effect of losing a given quantity of habitat increasing as the remaining habitat diminishes and hence also dependent on habitat changes in the more distant past [39,40]. Because different regions have been subject to anthropogenic pressures at different times [41], estimating the impact of contemporary changes in AOH thus necessitates information on each species’ likely AOH in the hypothetical absence of people (hereafter its ‘original’ AOH) [42].

**Figure 1.**
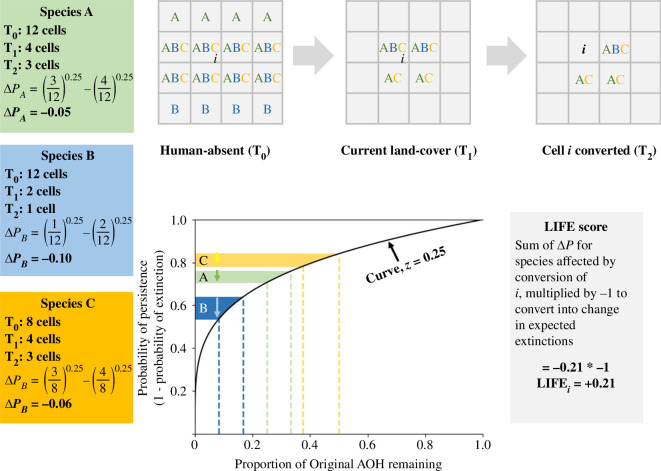
Illustrative example of calculating the change in probability of persistence across 100 years and the LIFE score associated with a change in land cover of a single cell (*i*), for the simple cases of three example species (A, B and C). Species A currently still has one-third of its human-absent AOH and loses 25% upon conversion of cell i. Species B has already lost a larger portion of its AOH, so the conversion of i has a greater impact on its probability of persistence than for A. The final species C demonstrates that not all species have the same historic range and the influence this has on changes in persistence. The LIFE score for the conversion of the cell is the sum of the changes in the probabilities of extinction (which is equal and opposite to the changes in their probabilities of persistence) for all species present in the cell. LIFE can be calculated in the same way for any land-use change including changes that result in increases in habitat such as restoration (see electronic supplementary material, figure S1).

The Durán *et al*. [19] method integrates original habitat extent and the non-linear impact of habitat loss on a species’ probability of persistence, assuming a power-law relationship between species remaining AOH and probability of persistence (i.e. of avoiding extinction) [11]. The probability of persistence is expressed as a function of the proportion of a species’ AOH remaining relative to its original AOH and so has a maximum value of 1 when a species occupies its original AOH (or indeed any larger area). Figure 1 illustrates the shape of this curve (assuming, for illustration, an exponent of 0.25) and the resultant change in probability of persistence of two hypothetical species when converting natural habitat in one cell. The shape of the curve means that if a species currently occupies a smaller fraction of its original AOH, the same absolute loss of AOH causes a greater reduction in persistence (Δ*P*; compare species A and B).

The Durán *et al.* [19] method can be used to estimate the change in probability of persistence resulting from retaining or restoring natural habitat in any area for each species whose global range and ecological requirements are known, and then summed across all species whose original AOH overlaps the area. Initial analyses applied this method to 1368 amphibian, bird and mammal species as well as 641 plants in the Brazilian Cerrado [19,43]. Because the metric is comparable across space, it was possible to estimate the changes in probability of persistence for all species as a consequence of sourcing soy from different parts of the Cerrado. Because results can be disaggregated, impacts on the probability of persistence of individual charismatic species (such as giant anteaters and jaguars) could also be derived. However, despite these scores having several desirable properties, their derivation for large numbers of species is computationally demanding, and so they have had limited uptake at global scale [24].

In this paper, we develop Durán’s [19] method into the LIFE metric by bringing AOH data for >30 000 terrestrial vertebrates together with high-performance computing to generate global, downloadable maps that summarize at 1 arc-min resolution the impact on the expected number of extinctions (either increases or decreases) of two archetypal land-cover changes: (i) converting natural habitat and pasture to arable, and (ii) restoration of current pasture and arable to their natural state. To align with the broad policy and societal focus on extinctions, we express the metric in terms of changes in probability of extinction (rather than persistence), but of course a change in extinction probability of a species is simply equal and opposite to that in its probability of persistence. Conversion to arable land was chosen because food and farming are responsible for more biodiversity loss than any other sector [5–7], and so maps of where agricultural impacts will be most acute are useful in guiding conservation and other decisions. We focused on restoration because of its high profile in international policy [44], including within the United Nation’s Decade on Ecosystem Restoration, and because mapping its potential impact provides information on where actions to reverse past habitat losses would be most effective. To better understand what the LIFE metric represents, for each of these mapped layers, we investigate how our scores vary with an area’s species richness, endemism and degree of habitat loss to date. Because the LIFE metric explicitly assumes non-linear relationships between habitat loss and extinction risk, we also examine its scalability—the extent to which scores derived for grid cells can be relied upon when actions are smaller or larger than those cells. Finally, we explore the sensitivity of our findings to different assumptions about how the probability of persistence responds to losses or gains of suitable habitat (i.e. to the shape of the persistence–AOH curve) and how far our results differ across major taxonomic groups. We begin, though, by explaining in detail how LIFE scores are derived.

## 4. Generating current and original areas of habitat

To derive global maps of the LIFE score for future land-cover changes, we first calculated current and estimated original AOH for all terrestrial vertebrate groups (amphibians, reptiles, birds and mammals) [4]. We did not include species with missing data, those that inhabit caves or subterranean habitats or those where mismatches between range maps, habitat maps and habitat preferences result in no measurable AOH either currently or in the past. We also exclude species that are listed as ‘marine’, ‘terrestrial + marine’, ‘freshwater’ or ‘freshwater + marine’ in the IUCN ‘systems’ field [4], which removes just over 500 species—largely penguins, marine mammals and sea snakes. This left us with 30 875 species (7188 amphibians, 8760 reptiles, 9447 birds and 5480 mammals). Current and original AOHs were generated for each species following Brooks *et al.* [35,45,46] (see Data accessibility). For current AOH, we used a map of the estimated distribution of habitats [47] in 2016. For the original AOH, we used a map of potential natural vegetation (PNV) [48], which estimates the distribution of habitat types in the absence of human impacts. The current layer maps IUCN level 1 and 2 habitats, but habitats in the PNV layer are mapped only at IUCN level 1, so to estimate species’ proportion of original AOH now remaining, we could only use natural habitats mapped at level 1 and artificial habitats at level 2. We overlaid these two habitat surfaces with species’ range maps from IUCN and Birdlife International and a Digital Elevation Model [49,50] and estimated AOH for each species’ range as those parts of its range that are (or were) suitable based on its elevation and habitat preferences (from IUCN) [4]. IUCN codes species’ range polygons based on species’ presence, origin and seasonality. We included those parts of a species’ range where its presence is ‘extant’ or ‘possibly extinct’, its origin is ‘native’, ‘reintroduced’ or ‘uncertain’ and the seasonal occurrence is ‘resident’, ‘breeding’, ‘non-breeding’ or ‘unknown’. When generating original AOH maps, we also included range polygons coded as ‘extinct’, acknowledging that these data are incomplete, particularly for amphibians. For species that exhibit seasonal habitat preferences, AOH was calculated separately for the breeding and non-breeding seasons.

**Figure 2.**
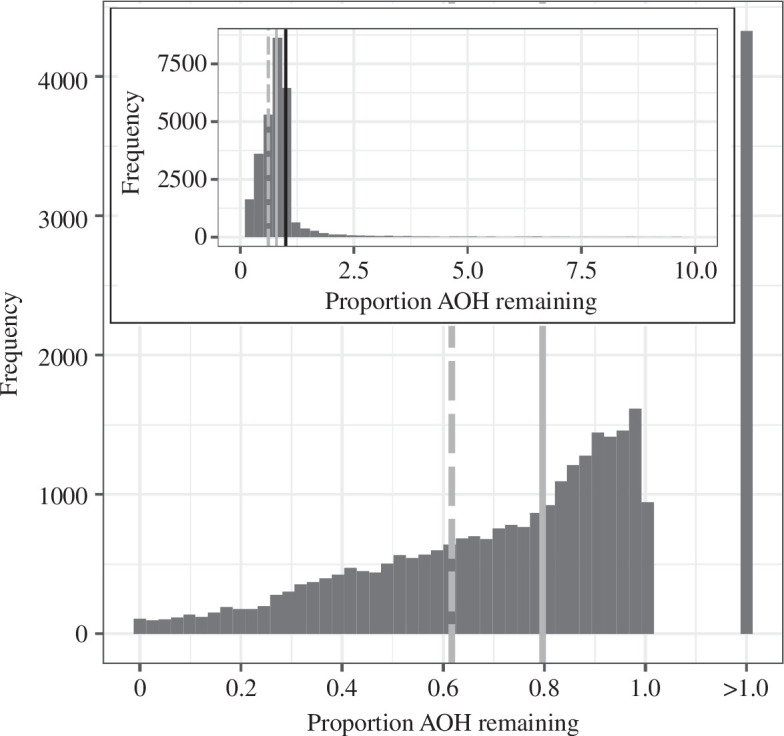
Histogram of the proportion of original AOH remaining for terrestrial vertebrate species (*n* = 30 875). For migratory species, we plot data only for the season with the lower value. Species with increased AOH as a result of human activity have values >1 and their distribution is depicted in the inset. The black line in the inset marks a value of 1. The geometric mean across all species and for those that have lost AOH as a result of human activity are depicted by solid and dashed grey lines, respectively.

For each species, we then calculated the extant proportion of its original AOH as the ratio of its current to original AOH (figure 2). This analysis indicates that 14.3% of species have a larger estimated AOH currently than in the absence of people, implying that human-mediated land-use change has enabled these species to expand their potential distributions, sometimes very substantially. Across all species, the geometric mean proportion of AOH remaining is 0.80. However, this figure is strongly influenced by very marked AOH expansions among some of those species that have apparently benefitted from human activity. Focusing instead on the 85.7% of species with smaller AOHs now than those estimated in the absence of people, their geometric mean proportion of AOH remaining is 0.62.

## 5. Using current and original areas of habitat to estimate marginal changes in probability of extinction

Following Durán *et al.* (2020), for each species, we then used our estimates of its current and original AOH to estimate the marginal impact of two contrasting sets of land-cover changes: the conversion of remaining natural habitats and non-urban artificial lands to arable land (our ‘conversion to arable scenario’) and the restoration of non-natural habitats (the ‘reversion to natural scenario’). In the conversion to arable scenario, all terrestrial habitats currently mapped as non-urban were converted to arable land. In the revert to natural scenario, all areas classified as arable or pasture were restored to their PNV (as mapped by [48]). In effect, here we are treating pasture as a semi-natural habitat that, despite often being actively managed, can still harbour substantial levels of biodiversity and therefore sits between natural and arable land. In both scenarios, land currently classified as urban was left unmodified because it is highly unlikely that either farmland expansion or restoration will encroach into existing urban areas. We estimated the effect on each species’ AOH of any conversion or reversion occurring in its current range polygon, even if that fell outside its current AOH—so under conversion, a species tolerant of arable could expand its AOH into previously unoccupied parts of its range, while under reversion, a species intolerant of cropland could expand back into restored natural habitat. Scenario-specific changes in AOH were calculated at the scale of 100 m pixels and then aggregated into 1 arc-min grid cells (approximately 1.86 × 1.86 km (3.4 km^2^ in area) at the equator) to facilitate downstream computation while still providing results at a fine enough scale to inform real-world decision-making.

Next, for each species, we translated the scenario-driven change in its AOH into a cell-specific change in its global probability of persistence and subsequently extinction risk over an appropriate relaxation period (following the approach summarized in §3 and in figure 1). The ‘true’ form of the persistence-habitat loss curve over a fixed period of time is of course not known and is likely to vary across taxa. To take a conservative approach and to avoid conjecture, we have based this iteration of LIFE on established literature, with a view to implementing improved persistence-AOH curves in the future. We followed previous studies using this approach by assuming an exponential function with an exponent of 0.25 [19,20,24,43], but we also tested the sensitivity of our broad findings to this assumption by using several alternative curve shapes (see §9). Because we were not concerned with those species with greater current than original population sizes (see §2), where the current or scenario estimate of a species’ AOH exceeded its original AOH, we capped its probability of persistence at 1 [20]. Following maps made by the IUCN for threatened species, we account for species occupying novel regions within the limits of their native range but not the colonization of areas beyond it, but note that new iterations of LIFE maps could be adjusted to include natural range expansions and assisted colonizations. For migratory species, probability of persistence in any scenario was derived separately for the species’ breeding and non-breeding ranges, with the overall change in persistence extinction risk for a given set of habitat changes then calculated as the difference between the geometric means of their breeding and non-breeding probabilities of persistence before and after the changes (based on equation 3 of Durán *et al*. [19]; see electronic supplementary material, section S4).

In the last stage, we summed the change in probability of persistence for all the species found in the cell. Significantly, this summed value of change in probability of persistence across species in a grid cell is numerically equal to the expected number of extinctions caused or avoided by conversion or reversion of that grid cell (for proof, see electronic supplementary material, section S3). To align with the broad policy focus on extinctions, we then multiply our persistence score values by −1 to convert them to changes in extinction risk. Finally, because the area undergoing change varies widely across cells, we divided the summed change in extinction risk scores by the area (in km^2^) of the cell restored or converted under that scenario to obtain an overall LIFE score describing the likely impact on the expected number of extinctions of converting or restoring 1 km^2^ of land. The scaling error associated with summing and then averaging 100 m pixel changes in this way is explored under §8 (Scalability).

## 6. Global maps of the LIFE score

**Figure 3.**
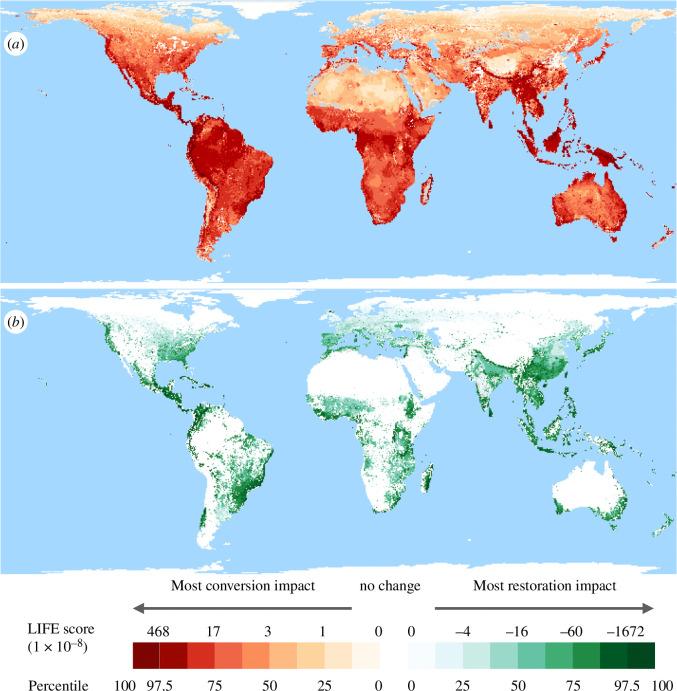
Global maps of LIFE scores associated with (*a*) conversion of remaining natural habitats to arable land and (*b*) restoration of cropland and pasture to natural habitats. The maps show changes in extinction risk realized over a 100 year time period summed across all study taxa (amphibians, reptiles, birds and mammals), aggregated to 1 arc-min grid cells and expressed as the average impact per km^2^ of conversion or reversion. Changes in probability of extinction are derived assuming a power-law persistence–habitat loss curve with *z* = 0.25. Break points divide the scores into octiles, except for the uppermost octile, where we also show the top 2.5% of most impactful land-use changes. Positive values indicate an increase in extinction risk, while negative values show a decrease in extinction risk. Negative values in the conversion map arise where species can inhabit arable, farmed land but not the natural habitat that it replaces. Conversely, positive values occur in the restoration map where species can inhabit farmed land but not the natural habitat that replaces it. Where land-use changes have the opposite from the predominant effect, they are shown in grey.

The LIFE score maps for our conversion and reversion scenarios (figure 3) prompt two overarching observations. First, while the per-km^2^ impacts on extinction of converting remaining habitats and pasture to arable land are very largely positive (indicating an increase in extinction risk) and those of restoring natural habitats very largely negative (indicating a decrease in extinction risk), the increases in extinction risk from conversion to arable tend to be both greater and more widely distributed than the decreases in extinction risk resulting from habitat reversion to natural. The relatively lower and patchier gains from reversion to natural arises because many grid cells currently have relatively little area under farming, and because to date there has been no conversion (at 100 m resolution) in some 1 arc-min grid cells of exceptional importance for vertebrate biodiversity. This overall comparison of the maps means that at the global scale we have far more to gain through habitat retention than through restoration. The importance of retaining existing natural habitats is underscored by the delayed and, in many cases, lower impacts of real-world habitat restoration compared with conversion [51,52]: any benefits plotted in our reversion surface are less clearcut and would likely take far longer to materialize than the increases in extinction risk shown in our conversion map.

A second observation is that for both scenarios, LIFE scores are highly skewed, with the majority of regions having relatively low values and a few regions scoring very highly. The conversion map highlights areas with high levels of vertebrate endemism, including several species-rich regions—such as the Guiana Shield, Cameroon, New Guinea and northern Australia—where to date clearance for agriculture has been relatively limited. Under reversion to natural, by contrast, the highest LIFE scores correspond to areas known to have large numbers of relatively narrowly distributed vertebrates that have already undergone extensive conversion to agriculture—including much of Brazil’s Atlantic Forest, eastern Madagascar, the highlands of Ethiopia and the Philippines. In the next section, we set out a more formal exploration of these spatial patterns.

## 7. Dissecting spatial variation in LIFE scores

To check our understanding of what LIFE scores represent, we investigated how well their spatial variation is predicted by three key components of the importance of land-cover change for global extinctions: species richness, the degree of endemism of the species present and the extent to which the species have already lost suitable habitat anywhere in their ranges. Because LIFE scores are summed across species, we anticipated that absolute values would covary positively with species richness. Because a unit area of land-cover change should have a greater impact on the probability of extinction of species with smaller global ranges, we expected absolute LIFE scores should be higher in grid cells whose species are on average more narrowly endemic. And because we consider that any given loss of AOH impacts more heavily those species that have lost more habitat already (figure 1), we expected positive associations between absolute LIFE scores and the average proportional loss of AOH to date of those species present.

To test these predictions, we calculated: richness as the number of species whose ranges overlapped a grid cell, endemism as the mean proportion of each species’ current total AOH made up by the cell and habitat loss to date as the mean proportion across each of its species of their original AOH that is no longer suitable for them (electronic supplementary material, figure S5). For the two scenarios (conversion to arable and reversion to natural), we focused on LIFE values with the predominant effect (i.e. positive LIFE scores associated with conversion and negative LIFE scores associated with reversion). The absolute value was taken for reversion values. We then modelled our log_10_-transformed LIFE scores in relation to these three predictor variables, including a spatial smoothing function for geographic location, by randomly sampling 170 000 cells (0.32 and 0.96% of the data for conversion and reversion, respectively) without replacement and calculating mean standardized effect sizes across 200 independent runs. Conversion and reversion impacts were modelled separately, only considering losses and gains, respectively, in each.

These analyses confirmed our understanding of what is captured in LIFE scores (table 1). Absolute values associated with conversion and reversion were greater for grid cells with higher species richness of terrestrial vertebrates, cells whose species on average exhibit greater endemism and cells whose species have already lost more of their original AOH. Standardized effect sizes were greatest for endemism, but all had relatively narrow confidence intervals across independent model runs. The modelled deviance explained ranged across runs from 79.4 to 89.6% and 69.1 to 76.4% for conversion and reversion, respectively.

**Table 1.** Mean standardized effect sizes and 95% confidence intervals for predictors of LIFE scores from our conversion and restoration scenarios. Effect sizes are from linear models fitted with a spatial smoother that used standardized log_10_-transformed values of the response and predictor variables. Negative LIFE scores from restoration were multiplied by −1 prior to log transformation.

land-cover change	predictor	mean	2.5%	97.5%
conversion to agriculture	endemism	1.106	0.809	1.287
	habitat loss to date	0.188	0.078	0.302
	species richness	0.705	0.548	0.845
reversion to natural	endemism	0.603	0.453	0.763
	habitat loss to date	0.448	0.346	0.554
	species richness	0.113	0.033	0.264

## 8. Scalability of LIFE scores

A central conceptual premise of the LIFE framework is that the relationship between a species’ remaining AOH and its probability of persistence is non-linear. This means that the per km^2^ impact on extinction risk of an action that is larger than the grid cell size at which an impact is computed is not exactly the same as the average across all affected grid cells and that of a smaller action is not the same as that of the entire grid cell that overlays it. However, running bespoke extinction risk calculations at the scale of any specific action would be impractical for most end-users, so instead we ran two sets of simulations to examine how far ‘true’ LIFE scores derived at exactly the scale of an action deviate from those estimated simply from using our existing 1 arc-min results. This deviation will depend on each species’ proportion of AOH remaining, the shape of the persistence–habitat loss curve and the size of the action.

Spatially modelling hundreds of actions was computationally prohibitive, so to test the scalability of our maps, we opted for a non-spatial statistical-modelling approach focused on the following five regions: South America, sub-Saharan Africa, south-eastern Asia, western Europe and northern Asia (Russia and Mongolia). For each region, we calculated the proportion of AOH remaining for each species present. To examine the scalability of our mapped LIFE scores for actions larger than our grid cells, we modelled 1000 actions across geometrically distributed sizes, ranging from the native resolution (3.4 km^2^ at the equator) to 10 million km^2^. For each action, the probability that a species was affected was governed by the portion of its AOH overlapping the region and the area of the action. The appropriate number of grid cells for the action size was then iteratively scattered across the region without replacement, with each having a chance to hit a given species. This procedure is essentially equivalent to assuming a homogenous random distribution of species within the region. Then, for each species, we calculated the ‘true’ impact of the simulated land-cover change and, derived from the grid cell values, expressed this deviation as a fraction of the ‘true’ value and summed these relative deviations across all species. We repeated the process for a total of 100 actions of each size.

**Figure 4.**
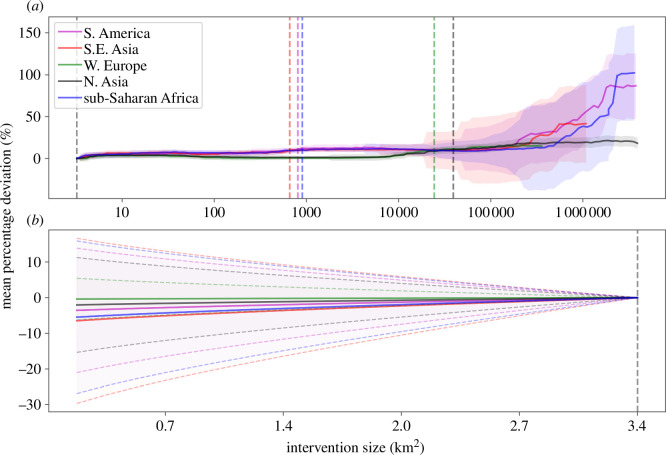
The modelled mean deviation (solid line) from the mapped LIFE scores for (*a*) actions that affect multiple pixels, with the 10% threshold marked by a dashed line, and (*b*) actions covering only a fraction of a pixel, with dashed lines marking the standard error ranges.

These simulation exercises suggested that our mapped surfaces can be used to impute the approximate per-km^2^ impact on extinctions of actions ranging up to 1000 km^2^ in size. Figure 4*a* shows how the summed relative deviation between the true and grid cell-derived values varies with action size. In western Europe and northern Asia, the incurred error remains <10% for actions up to 30 000 and 40 000 km^2^, respectively. South-east Asia, South America and sub-Saharan Africa reach 10% mean deviation at just under 1000 km^2^. These regional differences reflect the fact that species at lower latitudes have on average lost a greater proportion of their AOH already.

Adopting a similar modelling approach to test the validity of using mapped LIFE values for actions that are smaller than our mapped grid cells, we used the same sets of species and modelled 100 actions ranging in size from 0.05 to 1 arc-min on the side (0.17–3.4 km^2^ at the equator). When calculating the ‘true’ value of the simulated land-cover change, the area that the action alters within the grid cell is known and is added or removed from each species’ current AOH as appropriate. The fractional value, on the other hand, is calculated by multiplying the average LIFE score per unit area in the cell (which assumes land-use changes across the entire cell) by the area of the action. Figure 4*b* shows the results of this process. The mean summed deviation between the ‘true’ and grid cell-derived value remains low right down to 0.05 arc-min actions (where it reaches ~7% in SE Asia and less elsewhere). However, here the uncertainty in this deviation is ±25%, so we advise caution when using grid cell values for very small actions.

With these results in mind, we are confident that the LIFE surfaces presented in figure 3 can be used to evaluate changes in the statistically expected number of extinctions driven by land-cover changes of up to about 1000 km^2^. This does not preclude the use of LIFE as a means to assess larger changes, but doing so will incur a higher level of uncertainty or else bespoke calculations of LIFE, tailored to specific interventions (see Data accessibility). Of course, LIFE metric values are representative of a snapshot of the current state of global land cover, which is subject to change. This is also true of all other biodiversity metrics that consider land cover. Therefore, to minimize the risk of inaccuracies in the LIFE metric, it will be important to make use of the best and most recent land-cover data as and when it becomes available, especially in regions undergoing large-scale, rapid land-cover change.

## 9. Sensitivity analyses

We tested the sensitivity of spatial variation in LIFE scores to (i) the assumed shape of the relationship between a species’ probability of persistence and its loss of AOH, and (ii) what groups of species are included in the analysis.

### (a) Sensitivity to changing the persistence–habitat loss curve

The relationship between incremental losses of a species’ habitat and its risk of extinction is unknown and likely to vary widely across species: modes and rates of reproduction and dispersal, evolutionary history and vulnerability to other threats may each shape how species’ populations respond to anthropogenic habitat loss [53]. For our main analyses, we followed other studies [19,20,24,43,54] in assuming all species exhibit an exponential persistence–habitat loss curve with an exponent of 0.25, but we also explored how our two LIFE score surfaces differed using exponential curves with exponents set to 0.1, 0.5 and 1.0 (the latter indicating a linear response to habitat loss) and assuming probability of persistence changes according to a modified Gompertz curve (which allows for the disproportionate impact of stochasticity on persistence at low AOH values; see electronic supplementary material, figure S2 for curve shapes).

The LIFE score maps generally pick out the same broad regions of the world as being important for conversion and reversion regardless of curve specification—typically species-rich parts of the tropics and subtropics (electronic supplementary material, figure S6). However, comparison of the maps also shows, as might be expected, that assuming higher exponents tends to increase the homogeneity of LIFE scores: the impact of a unit AOH conversion (or reversion) becomes less sensitive to how much habitat conversion has already taken place. At the extreme, if persistence responds linearly to reductions in AOH (i.e. *z* = 1.0), the loss of a given AOH has the same impact regardless of how much of a species’ AOH has already cleared. If the assumption of a linear fit is biologically inappropriate [39], it thus risks underestimating the impact of losing (or restoring) the last remaining areas of habitat in highly converted regions while overestimating the impact of changes elsewhere. Conversely, maps generated with *z* set to 0.1 and especially those assuming a modified Gompertz relationship show greater spatial variation in LIFE scores and suggest the impacts of restoration or conversion would be relatively greater in regions that have already undergone extensive habitat clearance. We also conducted a simple variance analysis of the curve exponents and the Gompertz curve by comparing the variance of the scores within each pixel when different curve shapes were used. Most cells (77%) had a variance of less than 1% from the *z* = 0.25 curve. There was a higher level of variance in areas with a greater number of species, up to approximately 60% of the *z* = 0.25 value. This variance did not strongly correlate with the score itself (*r* = 0.0001).

### (b) Variation across taxonomic groups

Disaggregating LIFE scores by major taxonomic group (amphibians, reptiles, birds and mammals) again suggested our metric is broadly robust at a coarse scale (see electronic supplementary material, figure S7): for each of our taxonomic groups, the same regions would generally experience marked (and others, negligible) changes in species extinction risk following habitat conversion or reversion. However, there are some interesting differences when amphibians or reptiles are considered in isolation. Compared with all terrestrial vertebrates combined, for amphibians, land-cover changes in eastern North America and southern Europe are more impactful, while for reptiles, changes in some arid regions (such as the Sahara and central Australia) appear more important and those in higher latitude regions less important. These observations underscore the importance, in subsequent work, of expanding the LIFE metric to include additional taxa, most obviously any sizeable plant or invertebrate groups for which range maps and habitat preferences become available for a large proportion of the world’s species.

## 10. Overview, limitations and applications

By combining data on ranges and habitat preferences for 30 875 species of terrestrial vertebrates together with maps of the current and estimated original extent of habitat types, we generated two global, 1 arc-min resolution LIFE surfaces describing the present-day impacts on probable number of extinctions of converting or restoring natural habitats worldwide. Assuming species’ probability of persistence responds exponentially to changing AOH (with a *z*-value of 0.25; figure 1), habitat restoration would be particularly valuable per unit area in endemic-rich regions that have undergone extensive habitat clearance already (such as the Atlantic Forest, eastern Madagascar and the Ethiopian Highlands). Habitat retention, on the other hand, would have most impact in mitigating extinction in these regions too, but also in endemic as well as species-rich regions where there has been less marked conversion to date (such as the Guyana Shield, southeast Amazon Basin, Cameroon, eastern Congo, Greater Sundas and northern Australia). Statistical modelling of spatial variation in LIFE scores confirms these patterns, with impacts from conversion and restoration both co-varying positively with endemism, with the extent to which species have already lost AOH, and (especially for conversion scores) with species richness. We note that statistical exploration of variation in biodiversity metrics is unusual, but suggest similar formal interrogation of spatial patterns would be helpful in interpreting other global metrics as well.

In terms of the desirable characteristics of biodiversity metrics outlined above, LIFE scores have been devised to be directly comparable across space, such that a unit increase (or decrease) in summed probability of extinction reflects the same impact on the expected number of extinctions regardless of where it occurs. Our investigation of the scalability of LIFE scores suggests in addition that, despite our premise that habitat loss impacts species’ persistence in a non-linear way, the values presented in our 1 arc-min resolution surface can provide reasonably reliable estimates of impacts on extinction risk of land-cover changes ranging from 0.5 to 1000 km^2^. Our breakdown of findings by taxon illustrates that LIFE scores can be readily disaggregated according to the interests of the user. However, the resulting differences in LIFE score maps among major taxa make clear our vertebrate-only surface is not representative of terrestrial biodiversity as a whole, and so underline the importance of adding data on other groups as these become available. To be usable, such information needs to include the range and habitat preferences of all species in a taxon (or life form, such as trees)—across the entire area of interest. In the absence of such data, LIFE scores should be treated cautiously, especially in regions (such as Mediterranean biomes and the Cerrado) with higher relative richness and endemism among non-vertebrate groups [55].

The LIFE framework has several other limitations. Here we discuss five, the first two of which are linked to its underlying assumptions. First, as with any metric relating land-cover change to extinction risk, we lack a robust understanding of how species’ probability of persistence decreases as their AOH is reduced. Clearly, more work is needed to establish plausible curve shapes and explore how they are likely to vary across and within different groups of species. Reassuringly, we found broadly similar geographical variation in LIFE scores for exponential curves using *z*-values varying from 0.1 to 0.5, but a modified Gompertz curve resulted in markedly sharper geographical variation. The observation that a *z*-value of 1.0 produces somewhat more muted differences in apparent impacts suggests that assuming—as many metrics implicitly do—that extinction scales linearly with habitat loss [5,36,38,56] risks underestimating the potentially grave impacts of continued habitat conversion in already heavily converted regions.

Second, at present, the LIFE method treats all habitats listed as suitable by the IUCN as being of equal value to a species—ignoring differences in whether a habitat type is suitable or marginal for a species (because this information is currently only reported for 11% of species and because we lack information on what this difference in suitability means for species in terms of occupancy). This simplification assumes that population density is equal across different habitats within the species’ AOH, potentially overestimating the importance of marginal populations at range limits (and vice versa). Likewise, to date, LIFE also ignores effects of habitat patch size, fragmentation, connectivity, degradation and, critically, the impacts of other threatening processes (such as overexploitation or invasive species) that may limit a species’ ability to make use of otherwise suitable habitat [57]. These oversimplifications mean our scores overestimate the relative impact of habitat loss or restoration for species and places that are particularly affected by such processes. Likewise for those species able to live in agricultural land, our extinction risk scores take no account of differences in how that land is managed and hence underestimate the benefit of restoring areas currently subject to particularly damaging practices. Conversely, we take a conservative approach and, in line with the IUCN, do not allow species’ to colonize newly suitable areas outside of their current ranges, potentially underestimating the value of restoration [5]. We hope to address each of these simplifications in how LIFE deals with habitat suitability in future work.

Third, although our results on proportional losses of AOH (figure 2) align with a recent assessment that only around one-half of the area of ice-free biomes is still in areas of low human impact [58], our results are clearly only as reliable as the underlying data on species’ ranges, habitat preferences and habitat maps. Information is poorer for certain taxa and regions [59,60], and the natural habitat preferences for some species (including some nowadays exclusively associated with anthropogenic land uses) are entirely unknown. Estimates of species’ distributions in the absence of people are poor for many taxa, and this means that where we underestimate them and hence species’ habitat loss to date, LIFE scores will underestimate the effects both of further conversion and of restoration (because species are in reality further along the habitat loss trajectory than assumed). Work is in progress to ensure that the pipeline for calculating LIFE scores is readily updatable as new data on species and land-cover distributions become available (see Data accessibility). This is also important because the LIFE surfaces represent a snapshot of extinction risks today and should be updated periodically to reflect the changing availability of habitats, especially in regions of rapid conversion.

Fourth, we do not incorporate time lags between habitat change and biodiversity impact. In the case of conversion, we ignore extinction debts, and in the case of restoration, we do not consider delays or indeed uncertainties in species’ colonization and recovery. Caution is thus needed when comparing LIFE scores between our two maps. Values for restoration should certainly not be viewed as equivalent to those for conversion, and efforts to make comparisons—for instance, to inform offsetting activities for mitigating habitat damage—should employ explicit and conservative adjustment ratios to account for the much slower and less certain course of habitat restoration (see [5,52,61]); we suggest these ratios should be habitat- and region-specific.

Last, biodiversity metrics can be important in raising awareness of environmental change among the public and policymakers. Given this, it is important that metrics are easy to interpret [16]. Although the concept of extinction risk is relatively easy to communicate, the LIFE scores presented here are numerically small and not readily interpretable. Future developments should consider how to make these numbers more easily communicated—for example, by standardizing values relative to a chosen ‘average’ or ‘outstanding’ place in the world.

## 11. Conclusions

These significant caveats notwithstanding, we believe that the explicit consideration of the non-linear impacts of habitat loss and of long-term anthropogenic conversion, the transparent assumptions in the underlying method and the use of best-available data on almost 30 000 species mean the LIFE score is among the most powerful tools to date for quantifying the likely impacts on extinction of spatially explicit land-cover change. The LIFE layers are publicly available and can be easily combined with other data sources to assess the impact of land-cover changes across a broad range of actions, scales and geographies. For example, in terms of damaging activities, they can be linked in near-real time with remotely derived imagery to estimate and potentially attribute the extinction impacts of clearance events or wildfires. Combined with consumption and trade data, they can help assess the extinction footprint of specific products or businesses, the consequences of national trading decisions and even the impacts of individuals’ diets [43]. In terms of conservation actions, the LIFE layers can be used to estimate the effects of retaining or restoring particular areas of habitat and linked with cost data to help inform systematic conservation planning [62]. And at very large scale, they could be used to estimate the likely beneficial impacts of global-scale initiatives such as the recent international commitment to conserve 30% of land area by 2030—as well as (in combination with trade and economic data) to explore the likely negative effects such actions will have through displacing commodity production to other parts of the world. We welcome any such applications, as well as advice on how to improve the LIFE metric to make it more useful, accurate and representative.

## Data Availability

The LIFE surface data are provided in GeoTIFF format via [63] under the terms and conditions of the underlying species' elevation and habitat preference data and distribution polygons as laid out by the IUCN Redlist (https://www.iucnredlist.org/). Digital elevation maps are available from the USGS (https://earthexplorer.usgs.gov), and potential natural vegetation [48] and present land-cover maps [64] from their original sources. The LIFE pipeline source code is available under an open-source license (https://github.com/quantifyearth/life), allowing tailored impact analyses to be easily generated. Different land-cover maps and land-cover change scenarios may be examined, additional data may be used (such as better information on species occupancy) or users can focus on particular species sets of interest. The LIFE data can be easily regenerated as the underlying datasets are updated. We envisage our digital pipeline being regularly updated as upstream sources become available. All final statistical analyses for this manuscript were performed using the mgcv package (v1.9-0) in R (v4.3.2). IUCN data were processed using IUCN-modlib [65]. Supplementary material is available online [66].
